# Biochemical and Genetic Characterization of PspE and GlpE, Two Single-domain Sulfurtransferases of *Escherichia coli*

**DOI:** 10.2174/1874285800802010018

**Published:** 2008-03-18

**Authors:** Hui Cheng, Janet L Donahue, Scott E Battle, W. Keith Ray, Timothy J Larson

**Affiliations:** Department of Biochemistry, Virginia Polytechnic Institute and State University, Blacksburg, Virginia 24061 USA

## Abstract

The *pspE* and *glpE* genes of **Escherichia coli** encode periplasmic and cytoplasmic single-domain rhodaneses, respectively, that catalyzes sulfur transfer from thiosulfate to thiophilic acceptors. Strains deficient in either or both genes were constructed. Comparison of rhodanese activity in these strains revealed that PspE provides 85% of total rhodanese activity, with GlpE contributing most of the remainder. PspE activity was four times higher during growth on glycerol versus glucose, and was not induced by conditions that induce expression of the *psp* regulon. The *glpE/pspE* mutants displayed no apparent growth phenotypes, indicating that neither gene is required for biosynthesis of essential sulfur-containing molecules. PspE was purified by using cation exchange chromatography. Two distinct active peaks were eluted and differed in the degree of stable covalent modification, as assessed by mass spectrometry. The peak eluting earliest contained the equivalent mass of two additional sulfur atoms, whereas the second peak contained mainly one additional sulfur. Kinetic properties of purified PspE were consistent with catalysis occurring *via *a double-displacement mechanism *via *an enzyme-sulfur intermediate involving the active site cysteine. *K_m_*s for SSO_3_^2-^ and CN^-^ were 2.7 mM and 32 mM, respectively, and *k_cat_* was 64^s-1^. The enzyme also catalyzed transfer of sulfur from thiosulfate to dithiothreitol, ultimately releasing sulfide.

## INTRODUCTION

The enzyme rhodanese catalyzes the transfer of sulfur from thiosulfate to cyanide, producing thiocyanate and sulfite (for reviews, see [1,2]. Catalysis occurs via a ping-pong mechanism, with an active site cysteine residue of the enzyme carrying the transferred sulfur as a covalent intermediate (a cysteine persulfide). In the absence of cyanide, the covalently bound sulfur may be transferred to other thiophilic acceptors, such as thiols. When a dithiol is the acceptor substrate, sulfide and the oxidized disulfide are the products of the reaction. Bovine rhodanese was discovered first, and a variety of enzymological and structural studies have been performed on that and other similar enzymes [3-5]. The enzyme is comprised of two domains (~120 amino acids each) of similar structure that have limited amino acid sequence similarity to one another. The active site cysteine is only present within the carboxy-terminal rhodanese domain. Depending on the nature of the amino acids present in the active site loop, the tandem domain enzymes are classified as thiosulfate:cyanide sulfurtransferases, or as mercpatopyruvate sulfurtransferases [4].

Although rhodanese was discovered more than 70 years ago, physiological functions of various rhodaneses are just now being elucidated. Since the enzyme uses thiosulfate to convert toxic cyanide to the less toxic thiocyanate plus sulfite, it has been proposed that rhodaneses participate in cyanide detoxification [2,6,7]. It is likely, however, that proteins containing a rhodanese domain play a variety of roles in trafficking of sulfur (and selenium) during synthesis of other cellular metabolites. Genome sequence information reveals that almost all organisms encode multiple rhodaneses, some of which are tandem-domain rhodaneses. Other proteins consist of only a single rhodanese domain, and are fully capable of catalysis [8,9]. Finally, the rhodanese domain is predicted to be fused to a variety of other proteins. Knowledge of the functions of fusion partners, as well as of the genomic context of rhodanese-encoding genes in bacterial genomes has revealed the functions of some of the rhodanese domains. For example, the ThiI protein of *Escherichia coli* (needed for thiamin biosynthesis and for insertion of sulfur into uridine of certain tRNAs) contains a C-terminal rhodanese fusion, which was found to be essential for incorporation of the sulfur atom into 4-thiouridine of tRNAs [10,11]. Another rhodanese fusion, human MOCS3, contains a C-terminal rhodanese domain that participates in sulfur transfer during molybdopterin biosynthesis [12,13]. Bacterial MoeB/ThiF proteins correspond to human MOCS3 and are found with or without a C-terminal rhodanese domain, depending on the organism and metabolic system. It is possible that a rhodanese is generally needed for biosynthesis of molybdopterin and certain other sulfur-containing molecules. MoeB/ThiF proteins lacking the rhodanese domain may require a stand-alone rhodanese protein to provide sulfur transfer function.

Knowledge of the genomic context can often provide clues regarding function. YbbB of E. coli contains an N-terminal rhodanese domain and a C-terminal domain with an ATP-binding motif. In some organisms, ybbB is present within the selD operon. selD encodes selenophosphate synthetase, suggesting that ybbB might also be involved in selenium metabolism. It was found that the rhodanese domain of YbbB is necessary for incorporation of selenium into tRNA. YbbB catalyzes the conversion of 2-thiouridine to 2-selenouridine at the wobble position of certain tRNAs [14]. There are other examples where a rhodanese gene is found within an operon or cluster of genes known to be involved in sulfur metabolism. For example, the pathways for biosyntheses of pyridine-2,6-bis(thiocarboxylate) in Pseudomonas stutzeri and thioquinolobactin in P. fluorescens are dependent on enzymes similar to those involved in molybdopterin biosynthesis, and the gene clusters encoding these enzymes also encode a rhodanese [15,16]. Therefore, it is likely that these rhodaneses participate in sulfur transfer in a manner analogous to the rhodanese domain fused to the C-terminus of human MOCS3 [12].

In addition to thiI and ybbB, the genome of E. coli contains seven other genes that are predicted to encode proteins with one or more rhodanese domains. Four of them (yceA, ygaP, yibN and ynjE), or their corresponding proteins, have not been studied. The other three proteins (GlpE, PspE and SseA) have known enzymatic functions and have been characterized in some detail [4,8,9,17,18], but their physiological functions are not known. GlpE and PspE are single-domain rhodaneses, and SseA is a tandem-domain mercaptopyruvate sulfurtransferase. GlpE is encoded by the first gene in the complex glpEGR operon [19], which also encodes a rhomboid protein (GlpG [20]) and the repressor (GlpR) for the glycerol 3-phosphate regulon [21]. This genomic context suggests that a rhodanese might participate in the regulatory functions exerted by GlpG or GlpR, but it was found that the complex glpEGR operon is not subject to regulation by the glpR-encoded repressor, placing in doubt the possibility that GlpE plays a role in the utilization of glycerol 3-phosphate. PspE, a periplasmic protein, is reported to be a member of the pspABCDE (phage shock protein) operon [22,23]. Yet it is not clear what role a periplasmic sulfurtransferase might play in the cellular stress response to filamentous phage infection or other stresses, such as high salt, heat shock, or organic solvents, which are also known to induce this operon [24,25].

In the work reported here, genetic and biochemical approaches were used to show that glpE is apparently dispensable for function of the glycerol 3-phosphate regulon. In addition, neither glpE nor pspE are essential for production of sulfur-containing cofactors or amino acids. Finally, the pspE gene does not appear to be coregulated with other members of the phage shock operon, but is regulated by the nature of the carbon source present in the growth medium.

## MATERIALS AND METHODOLOGY

### Materials

Unless specified otherwise, the reagents used were of highest quality feasible, generally obtained from New England Biolabs, Sigma-Aldrich or Fisher Scientific.

### Bacterial Strains

The bacterial strains used or constructed are described in Table **1**. Strain DH5αZ1 was used as a host for construction of recombinant plasmids.

### Media and Growth Conditions

Cultures were grown aerobically at 37^o^C in Luria-Bertani broth (LB) [26] in the presence of antibiotic as appropriate (100 μg/ml ampicillin (Ap), 25 μg/ml kanamycin (Km) or 10 μg/ml tetracycline (Tc)). For minimal medium, M9 salts [26] were supplemented with 5 μg of thiamin per ml, 0.4% glucose or glycerol as indicated, and the appropriate antibiotics. For testing of potential auxotrophies, cultures were grown overnight in liquid minimal medium containing low levels of supplements, harvested, washed four times with medium lacking the vitamin to be tested, and then diluted 500 fold into minimal medium lacking the vitamin [27]. Growth was followed by monitoring optical density for 24 h. To test the ability of sulfurtransferase mutants to synthesize molybdopterin, mutants were cultured anaerobically on LB containing 20 mM sodium chlorate. Molybdopterin-deficient strains are chlorate resistant, since they are unable to assemble active nitrate reductase [28]. Nitrate reductase is capable of converting chlorate to chlorite, which is toxic. For all phenotypic testing, control strains harboring mutations in one of the known biosynthetic genes were used as controls.

### Construction of Plasmid pFRT-K and pSB1

A Km^r^ cassette, flanked by directly repeated FRT sites, was obtained from pCP15 [36] and cloned as an *Eco*RI-*Hin*dIII fragment into the same sites of pBluescript-KS^+^ to create pFRT-K [37]. Plasmid pPS-3, provided by Peter Model (The Rockefeller University) contains the *psp* operon on a 4.5 kb *Eco*RI fragment in pBluescript. The *pspE* gene was subcloned from pPS-3 [38] as a 723 bp *Sal*I–*Eco*RI fragment into the same sites of pBluescript-KS^+^ to create pSB1.

### Deletion of the Chromosomal *pspE* Gene

A plasmid in which most of the *pspE* gene was replaced by a Km^r^ FRT cassette was constructed by cloning DNA fragments that flank *pspE* into pFRT-K such that these regions flank the Km^r^ FRT cassette. The 5’ flanking (412 bp on a *Xho*I-*Dra*I fragment) and 3’ flanking regions (115 bp on a *Psh*AI-*Bam*HI fragment) of *pspE* were obtained from pSB1 and cloned into the *Xho*I-*Hin*cII and *Sma*I-*Bam*HI sites of pFRT-K, respectively, yielding pHCE2 in which codons 3-71 of *pspE* gene were deleted. The method of Datsenko and Wanner [35] was used for replacing the wild-type *pspE* allele of strain BW25113(pKD46) with the *ΔpspE*::Km^r^ *FRT* allele of pHCE2 to create strain HC1.1. Using bacteriophage P1-mediated transduction [34], *ΔpspE*::Km^r^ *FRT* was moved into strain TL524 for testing of potential phenotypes (strain HC6.1). A kanamycin-sensitive derivative of HC6.1 (named HC7.1) was obtained by Flp-mediated excision of the Km^r^ cassette of *ΔpspE*::Km^r^ *FRT* using pCP20 [36]. The *pspE* deletion/replacement alleles of both strains were verified by PCR analysis using appropriate primers and chromosomal DNA of strains HC6.1 and HC7.1 as templates. In addition, the Km^r^ cassette was mapped to the expected chromosomal location by demonstrating that it was 98% cotransducible with the *zcj-233*::Tn*10* allele of strain CAG12028 during phage P1-mediated transduction. This Tn*10* is located within 1200 bp of *pspE *[32].

### Deletion of the Chromosomal *glpE* Gene

The *glpE* gene was deleted in a manner analogous to that described above for *pspE*. Plasmid pJAB1 was constructed with appropriate restriction fragments cloned on either side of the Km^r^ FRT cassette of pFRT-K such that 204 bp of *glpE* were replaced, beginning at the *Bst*BI site within codon 5 of *glpE* (nucleotides 3559629-3559833 of the *E. coli* chromosome, GenBank LOCUS NC_000913; details available upon request). *E. coli* host strain BW25113(pKD46) was transformed with linearized pJAB1 with selection for kanamycin resistance. The position of the *ΔglpE*::Km^r^ *FRT* allele of one transformants, strain AL1, was found to be at the expected location by P1-mediated cotransduction with the *malT*::Tn*10* allele of strain TST3 (70% cotransduction observed). The *ΔglpE*::Km^r^ *FRT* allele of AL1 was moved by P1 transduction into strains TL524 and HC7.1 by selection for kanamycin resistance, resulting in strains JLD17201 and HC8.1, respectively. The Km^r^ cassette was excised from these strains by FLP-mediated recombination [36], resulting in strains JLD17204 and FA035 (Table **1**). The *ΔglpE*::Km^r^ *FRT *and *ΔglpE*::*FRT* alleles were verified by PCR.

### Construction of PspE Overexpression Plasmid pHC4.1

The *pspE* gene was amplified from genomic DNA of strain MG1655 by PCR using the primers pspE1H1, ccatagaaggacgctCaTatgtttaa and pspE2H1, cgctcatggtgaattctt*tta*a cct (stop codon in italics), where the underlined letters indicate restriction sites for *Nde*I and *Eco*RI, and the uppercase letters are mismatches. After cleavage with *Nde*I and *Eco*RI, the amplified product was cloned into the same sites of pT7-7 [30], yielding pHC4.1.

### Purification of PspE

Strain BL21(DE3)(pHC4.1) was grown in 750 ml of LB-ampicillin to an OD_600_ of ~0.5, and expression of PspE was induced for 2 to 4 h by the addition of 0.5 mM IPTG (OD_600_ of ~1). Cells were harvested (4 ^o^C) by centrifugation and washed with 30 ml of 20 mM sodium acetate, pH 5 (buffer A). The cell pellet was stored at –70 ^o^C. Freeze-thaw extraction of PspE was based on the same method used for purification of GlpE [8]. Frozen cells were thawed on ice, suspended in 15 ml of buffer A, and incubated on ice for 30 min. Cell debris was removed from the extract by centrifugation at 40,000 × g for 90 min. The freeze-thaw extract was loaded onto a prepacked Waters propylsulfonic acid (SP) cation-exchange column (1 × 10 cm) equilibrated at room temperature with buffer A. After extensive washing with buffer A, the column was developed with a 55-ml linear gradient of 0 to 825 mM NaCl. Two peaks containing rhodanese activity (PspE1 and PspE2), eluting between 330 and 600 mM NaCl, were pooled separately, adjusted to pH 7.0 by addition of 20 μl of 1 M Tris-HCl (pH 8.0) per ml of collected fraction, and stored in aliquots at 4 ^o^C.

### Polyacrylamide Gel Electrophoresis

Sodium dodecyl sulfate-polyacrylamide gel electrophoresis was performed as described by Laemmli [39] on 15% polyacrylamide gels.

Non-denaturing polyacrylamide gel electrophoresis was performed using the same gel and buffer system as above, but lacking SDS. The sample loading buffer contained 62.5 mM Tris-HCl (pH 6.8), 10% glycerol, 0.03% bromophenol blue. Gels were pre-electrophoresed for 1h.

### Assay of Sulfurtransferase Activities

The standard rhodanese assay was essentially that described previously to quantify thiocyanate [8,40]. Assays (0.5 ml) contained 100 mM Tris-acetate (pH 8.6), 10 mM ammonium thiosulfate, 50 mM KCN, and enzyme. Reactions were initiated by the addition of KCN and terminated, after 2 min, by the addition of 0.25 ml of 15% formaldehyde, followed by 0.75 ml of ferric nitrate reagent. The absorbance at 460 nm was determined and compared to a standard curve for thiocyanate. One unit of activity is defined as the amount of enzyme that catalyzes the production of 1 μmol of thiocyanate per min at 25 °C. For kinetic studies, the concentration of the Tris-acetate buffer was increased to 200 mM, which helped maintained constant pH as substrate concentrations were varied.

Standard assays (1-ml) employing dithiothreitol as sulfur acceptor contained the same components as above except that 10 mM dithiothreitol replaced KCN. Absorbance at 283 nm was monitored continuously and activity was calculated based on an extinction coefficient of 273 M^-1^ cm^-1^ for oxidized dithiothreitol.

Mercaptopyruvate sulfurtransferase activity catalyzed by PspE was assayed using two methods. The first was a modification of the assay used for rhodanese with substitution of 50 mM mercaptopyruvate for thiosulfate. Reactions were initiated by the addition of enzyme and terminated after a 15-min incubation at 25 (C. The second assay used 10 mM 3-mercaptopyruvate as the sulfur donor and 25 mM 2-mercaptoethanol as sulfur acceptor. The production of pyruvate was measured following its reaction with 2,4-dinitrophenylhydrazine [41].

### Determination of Cyanolyzable Sulfur of PspE

Persulfide sulfur bound to cysteine residues of proteins can be quantitatively released by treatment with CN^-^ and quantified as SCN^-^ [42]. The two peaks of PspE obtained from cation-exchange chromatography were adjusted to pH 7.0 by addition of 1 M Tris-HCl pH 8.0, because persulfide sulfur is acid labile [43]. Various amounts of PspE were treated with 100 mM cyanide for 5 min at room temperature. Thiocyanate was then quantified as the ferric thiocyanate complex as described for the rhodanese assay.

### Analysis of Covalent Modification of PspE by Mass Spectrometry

Aliquots of PspE1 and PspE2 (20 – 50 nmol in 0.5 ml) eluted from the cation exchange column were thoroughly dialyzed against 10 mM Tris-HCl (pH 7.5). An aliquot of PspE2 was treated with 1 mM ammonium thiosulfate for 15 min at room temperature. The samples were prepared for mass spectrometry by precipitation with four volumes of ice-cold methanol (–70 °C, overnight). Precipitated proteins were washed with cold 70% methanol, dried, and stored at 4 °C. Protein pellets were suspended in 50% methanol, 10% formic acid at approximately 20 pmol/μL. Samples were analyzed using a Thermo Electron TSQ Quantum AM mass spectrometer equipped with an electrospray source. Injection was at 3-5 μL per min using a 250 μL Hamilton gas-tight syringe. Spray voltage was 4000 volts, sheath gas pressure was 10 psi, capillary tube temperature was 270 °C (tube lens offset was 160 and the lens offset was -1.3. The machine had been tuned and calibrated using caffeine as a standard). The m/z range from 550 to 1500 was scanned using Q1 with a 1.5 second scan time and a peak width of 0.7. Data were collected for approximately 5 to 10 min and the spectra were averaged using Xcalibur software. Deconvolution of the averaged spectra was performed manually.

## Results

### Isolation and Characterization of *pspE* and *glpE* Mutants

In order to assess the relative contributions of the two single-domain rodaneses to cellular sulfurtransferase activity, and possibly to cellular physiology, strains deficient in either pspE, glpE or both were constructed. In each case, the majority of the gene was ultimately replaced by the FRT scar. All insertion and deletion alleles were verified by PCR, and were mapped to the expected chromosomal locations by using phage P1-mediated cotransduction with an independent neighboring allele.

Growth phenotypes of the three mutant strains were compared with the wild type using rich and minimal media. All four strains were capable of growth on glucose, glycerol or acetate minimal media without supplementation. Growth on minimal medium indicated that neither glpE nor pspE is required for production of sulfur containing amino acids (cysteine and methionine) or cofactors (thiamin, biotin, lipoate). To find out if either gene is required for molybdopterin biosynthesis, the strains were plated on rich medium containing chlorate and incubated under anaerobic conditions. Under these conditions, strains unable to produce molybdopterin display a chlorate-resistant phenotype, since they are unable to produce active nitrate reductase (nitrate reductase converts chlorate to chlorite, which is toxic [28]). All four strains displayed equal sensitivity toward chlorate, indicating that pspE and glpE are not essential for production of molybdopterin.

The periplasmic location of PspE suggested that it might serve as a detoxification enzyme for cyanide or for heavy metal cations such as cadmium. However, the pspE mutant HC7.1 was not more sensitive to KCN (0-1 mM) when compared to the wild type during growth on LB. Addition of 1 mM thiosulfate had no influence on the shapes of the growth curves. The results suggest that PspE does not play a role in cyanide detoxification, and are in agreement with those of Adams et al. [9], who reported that overexpression of PspE did not confer resistance to cyanide.

The periplasmic Dsb (disulfide bond formation) proteins, DsbA and DsbB, are involved in Hg^2+^ and Cd^2+^ detoxification by an unknown mechanism [44,45]. Although the Dsb proteins generally function to maintain disulfide bonds in periplasmic proteins, the possibility that the flow of electron could be from periplasmic dithiol protein, through the Dsb system to the persulfide form of periplasmic PspE was considered. Reduction of the cysteine persulfide would result in formation of hydrogen sulfide, which would precipitate (thereby detoxifying) heavy metal cations at the cell surface. If this were the case, mutation of pspE would be predicted to increase the sensitivity of E. coli to Hg2+ and Cd2+. Growth of wild-type and mutant strains deficient in pspE was compared on solid LB agar containing various concentrations of CdSO4 (0.01, 0.03, 0.1, 0.3 and 1.0 mM). Except for medium containing 1 mM Cd2+, all strains grew in the presence of Cd^2+^. No significant difference between the sulfurtransferase-deficient strains and the wild-type strain was observed.

A variety of other phenotypic tests were performed in which growth of the rhodanese-deficient strains was compared to that of the wild type. These included sensitivity to oxidative stress (peroxide, methyl viologen, selenite), antibiotics (tetracycline, chloramphenicol, spectinomycin, ampicillin, streptomycin, kanamycin, nalidixic acid), and detergent (deoxycholate). The ability to utilize a variety of compounds (sulfate, thiosulfate, taurine, cysteine) as sole sulfur source at relatively low concentration (0.05 – 0.1 mM) was also investigated. In all cases, growth of the mutant strains was comparable to that of the wild-type strain.

### Rhodanese Activities in Wild-Type and *pspE/glpE* Mutant Strains

In order to determine the contributions of PspE and GlpE to total rhodanese activity in E. coli, activities in cell-free extracts of the wild-type and the single and double mutant strains were determined following growth in glucose minimal medium. The results indicate that the pspE gene is responsible for contributing most of the rhodanese activity (Table **2**).

In various experiments, it was found that deletion of pspE decreased total rhodanese activity by 80 – 90 %, whereas deletion of glpE resulted in a decrease of about 10 %. Only 5% of total rhodanese activity remained upon deletion of both genes. Residual activity could be due to the presence of one or more of the other proteins harboring a rhodanese domain.

### Regulation of *pspE* and *glpE* Expression

**Fig. (1):**
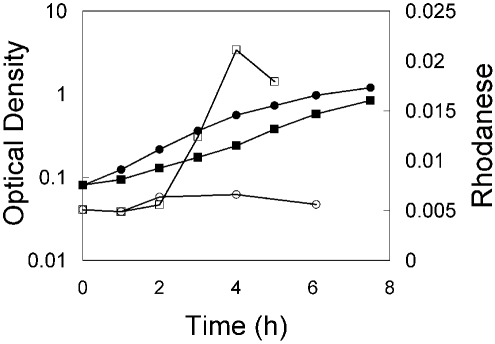
Rhodanese activities during growth on glycerol or glucose minimal medium. Strain JLD17204 (*ΔglpE::FRT*) was grown overnight in minimal medium with glucose. Cells were harvested by centrifugation, washed, and then diluted into medium with either glucose (circles) or glycerol (squares). The optical density at 600 nm (closed symbols) and rhodanese activity (open symbols) were monitored. Rhodanese activity was determined using intact cells harvested at the indicated time points and corresponds to the activity (units) found in the equivalent of 1 ml of cells at an OD of 2. Substrate concentrations of 50 mM thiosulfate and 50 mM cyanide were employed for these assays.

Clues regarding function of the sulfurtransferases of *E. coli* were sought by investigating global gene expression data available in ASAP [46]. These data indicated that *pspE* mRNA levels were expressed at higher levels during growth on glycerol, acetate or proline as carbon source relative to expression during growth on glucose. The expression of *glpE* was about the same regardless of carbon source. To determine if the apparent increased content of *pspE* mRNA during growth on glycerol minimal medium is reflected in an increase in rhodanese activity, an overnight culture of strain JLD17204 (*ΔglpE*) was diluted into fresh minimal medium containing either glycerol or glucose as carbon source. At timed intervals, aliquots were withdrawn for measurement of optical density and rhodanese activity. Rhodanese activity was induced four to five fold during exponential growth on glycerol relative to the level of activity found in cells growing on glucose (Fig. **1**).

**Fig. (2):**
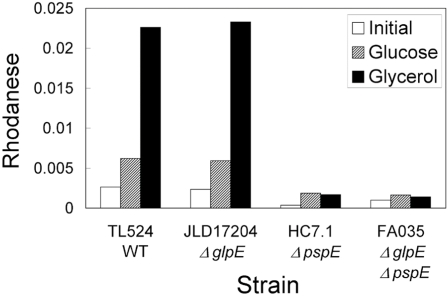
Rhodanese activity in wild-type and mutant strains. The indicated strains were grown overnight in glucose minimal medium, washed and then diluted into medium containing glucose (striped bars) or glycerol (black bars). Cells were harvested during exponential growth (OD600 of 0.64-0.79) and rhodanese activities were determined as described in the legend to Fig. (**1**). Activities found in the overnight cultures are indicated by the open bars.

Induction of rhodanese activity was due to an increase in *pspE* expression, since the phenomenon did not occur in the strains in which *pspE* was deleted (Fig. **2**).

The *pspE* gene is part of the *pspABCDE* operon, but apparently is also transcribed independently from the other genes of the operon [23]. The rhodanese activity of PspE provides a facile reporter for expression of the operon. To determine if *pspE* is induced in response to the stressors that induce other members of the *psp* operon, rhodanese specific activity was determined in cultures of strain JLD17204 (*ΔglpE*) growing in glucose minimal medium following various treatments (salt stress using 0.75 M NaCl or heat stress at 50 ^o^C for 10 min). No increase in rhodanese activity was apparent in aliquots taken at 20, 40, 60 and 135 min following addition of NaCl (relative to unstressed cells). Small (10% and 40%) increases in rhodanese activity were observed 20 and 40 min following heat stress, but then activity declined. These observations indicate that *pspE* is not significantly co-regulated with the other members of the *pspABCD* operon, whose expression increases much more dramatically following stress. Data available in ASAP suggest that *pspABCD* operon mRNA levels increase from 5 to 10 fold following heat stress, while *pspE* mRNA levels increase only 1.5 fold [47].

### Purification and Characterization of PspE

**Fig. (3):**
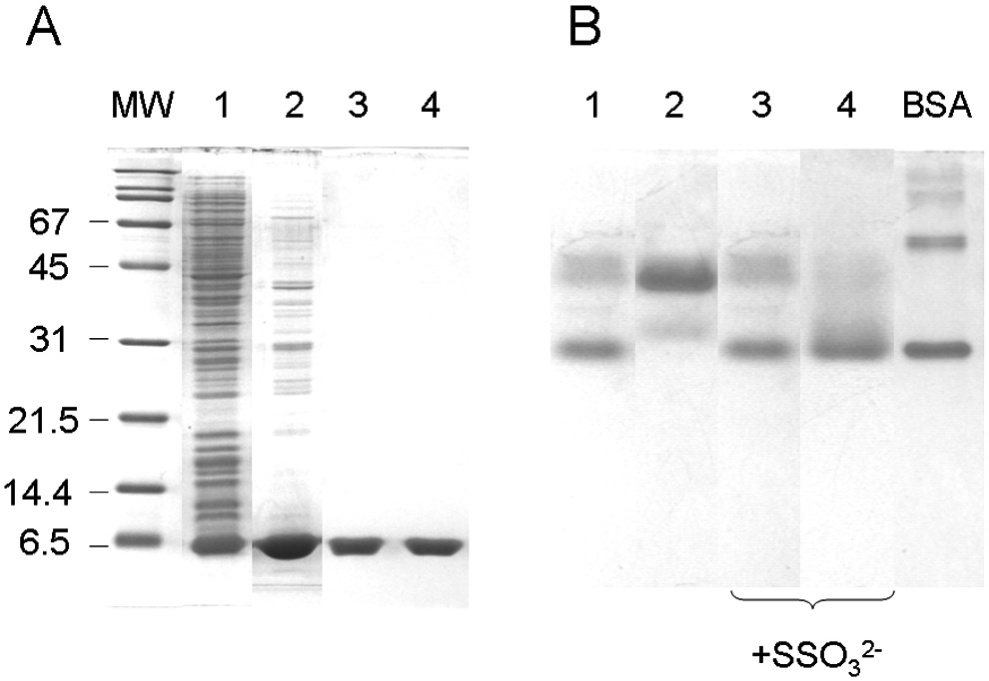
Electrophoretic analysis of PspE1 and PspE2. **A:** Fractions obtained during purification of PspE from induced strain BL21(DE3)(pHC4.1) were analyzed on an SDS-polyacrylamide gel. Lane 1, total cellular proteins (10 μg). Lane 2, freeze-thaw extract (10 μg). Lanes 3 and 4, pooled peak 1 (PspE1) and peak 2 (PspE2), respectively, from cation-exchange chromatography (3 μg 	each lane). MW, molecular weight standards. **B.** PspE1 (lanes 1 and 3) and PspE2 (lanes 2 and 4) were analyzed using nondenaturing polyacrylamide gel electrophoresis. In each lane, 3 μg of PspE was analyzed. Where indicated, PspE was pre-incubated with 1 mM ammonium thiosulfate for 5 min at 22^o^C. BSA, 2 μg BSA (monomeric and dimeric forms are the two major bands) was used as a standard.

PspE was previously purified as a cytoplasmically expressed protein possessing an N-terminal His-6 affinity tag [9]. Native PspE is synthesized as a precursor with a 19 amino acid N-terminal signal sequence and is secreted to the periplasm [38,48]. Mature PspE, comprised of only 85 amino acids, is the smallest known active rhodanese. In the present work, native PspE was overexpressed and purified as described in the “Methods.” Strong overexpression and its periplasmic location facilitated purification of PspE by freeze-thaw treatment, followed by cation exchange chromatography. A four-fold purification yielded approximately 5 mg of PspE from a 750-ml culture. Using Coomassie blue staining following SDS-PAGE, PspE was estimated to be >95% pure (Fig. **3A**), and had a specific activity of 130 – 150 U per mg of protein.

Elution of PspE from the cation exchange column using a salt gradient yielded two narrowly-separated but distinct peaks. The two peaks (PspE1 and PspE2) were collected separately and characterized. PspE1 had a more compact and/or more negatively charged form as analyzed by nondenaturing PAGE (Fig. **3B**) and by gel chromatography (not shown). Treatment of PspE2 with 1 mM ammonium thiosulfate resulted in its conversion to the faster migrating form observed in fraction PspE1 (Fig. 3B), suggesting that transfer of sulfur from thiosulfate to the active site cysteine of PspE2 results in its conversion to PspE1.

**Fig. (4):**
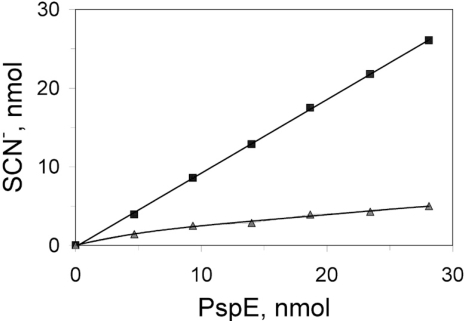
Cyanolyzable sulfur of PspE1 and PspE2. Immediately following elution from the cation exchange column, fractions containing PspE1 (■) and PspE2 (▲) were adjusted to pH 7.0 by addition of 1 M Tris-HCl (pH 8.0). Aliquots were then treated with 100 mM cyanide for 5 min at room temperature. Thiocyanate was quantified as the ferric thiocyanate complex as described for the rhodanese assay. The amount of PspE was estimated using the Bradford protein assay [50], with bovine serum albumin as standard.

There is only one cysteine residue in PspE. Evidence for covalent modification of the active site cysteine of PspE was obtained by using several methods. First, it was found that PspE1 contained approximately one equivalent of cyanolyzable sulfur, with much less sulfur present in PspE2 (Fig. **4**). Second, PspE1 was much more tolerant to chemical inactivation by the cysteine-modifying reagent 5,5’-dithio-bis(2-nitrobenzoic acid) (DTNB) compared with PspE2 (data not shown). It is known that the sulfur-free form of bovine rhodanese is twice as reactive toward DTNB relative to the cysteine persulfide form of the enzyme [49], and the same appears to be the case for PspE.

Finally, analysis of the two fractions by electrospray mass spectrometry revealed that multiple, covalently modified forms of the enzyme are present in the preparations. Based upon the properties of other rhodaneses, the data are most consistent with modifications occurring on the sulfhydryl group of the active site cysteine of PspE. PspE1 was more highly modified than PspE2 (Table **3**). In fact, the predominant species of PspE present in PspE1 contained the equivalent mass of either one or two sulfur atoms (along with minor amounts of more highly modified PspE). The predominant enzyme forms present in PspE2 were PspE with one sulfur atom, along with smaller amounts of unmodified PspE. A substantial fraction of both PspE1 and PspE2 apparently is present as the sulfonate-modified form (E-SSO_3_H). Treatment of PspE2 with thiosulfate resulted in greater abundances of the modified forms of PspE. The observation that PspE2 released less than one equivalent of sulfur upon treatment with cyanide even though the predominant form present was the cysteine persulfide suggests that the sulfur-modified form of the enzyme is quite stable to this treatment.

Analysis of PspE by mass spectrometry confirmed that it was processed properly during its secretion to the periplasm, even when expressed at high levels. The molecular masses observed matched exactly those expected if the 19 residue signal sequence was removed.

The question arises as to the source of the covalent modifications of PspE. Thiosulfate is sometimes added to the buffers during purification of rhodaneses [8,51]. This practice insures that the active site cysteine is modified, that is, converted to its persulfide form, and as such, is more resistant to oxidation. However, we did not include thiosulfate in the buffers used during purification, and so it is likely that PspE is covalently modified *in vivo*, and that such modifications are relatively stable. The modifications of PspE could arise from an endogenous metabolic pathway that yields the sulfur donor, or it is possible that the growth medium contains thiosulfate or other potential sulfur donors. To determine if growth medium contains a potential sulfur source, cyanolyzable sulfur was determined in LB medium. It was found that LB contains about 0.013 mM cyanolyzable sulfur by using PspE and a rhodanese assay run to completion. This sulfur could be the source of sulfur for modification of PspE prior to its purification by the standard protocol described above. The other possibility, that an endogenous pathway is responsible for production of a sulfur donor that modifies PspE, was tested by production of PspE during growth on a defined minimal medium that contains no added thiosulfate, but instead, only sulfate as sulfur source. PspE expression was induced during growth on M9 glucose minimal medium using strain BL21(DE3)(pHC4.1). It was found that the spent growth medium contained about 0.037 mM sulfur donor, as assessed by the PspE-catalyzed rhodanese reaction. Furthermore, PspE produced during growth on M9-glucose eluted as two peaks during cation exchange chromatography, the first of which contained cyanolyzable sulfur (data not shown). These results show that during normal growth, *E. coli* produces compounds that provide sulfur for covalent modification of PspE with sulfane sulfur.

### Catalytic Properties of Purified PspE

**Fig. (5):**
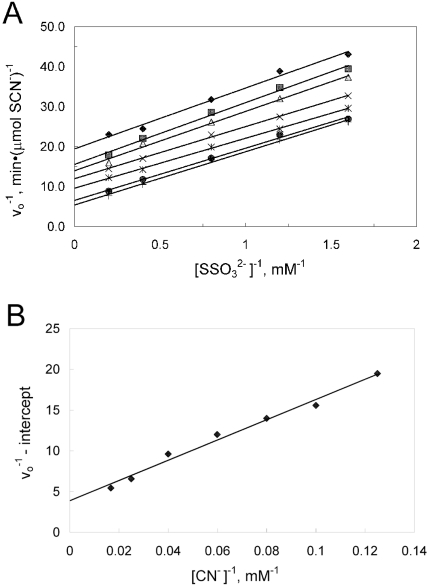
Kinetic characterization of the thiosulfate:cyanide sulfurtransferase reaction catalyzed by PspE. Each assay contained 0.63 μg of purified PspE2 and 1 mM cysteine. (A) Doublereciprocal plot of the rate of thiocyanate formation versus thiosulfate concentration at various fixed concentrations of cyanide: 8 mM (♦), 10 mM (■), 12.5 mM (▲), 16.7 mM (×), 25 mM (*), 40 mM (●), 60 mM (+). (B) Secondary double-reciprocal plot of apparent *V^max^* values from the data in panel A versus cyanide concentrations.

Purified preparations of PspE exhibited specific activities of 150 -200 U/mg when assaying transfer of sulfur from thiosulfate to cyanide. Rhodaneses utilize a double-displacement (ping-pong) mechanism [8,52], which is also apparently the case for PspE [9]. In the previous study, PspE contained an N-terminal His_6_ tag, which conceivably could affect the catalytic properties of PspE. Thus, kinetic properties of PspE were reinvestigated and alternate substrates were tested as sulfur donors/acceptors. PspE2 was used to study the kinetic properties in reactions containing 1 mM cysteine, which activates PspE activity. Data from activity measurements of purified PspE2 with various concentrations of thiosulfate at fixed concentrations of cyanide fit the equation describing a double-displacement mechanism. A plot of the data (Fig. **5**) yielded *K_m_* values for thiosulfate and cyanide of 2.7 and 32 mM, respectively. The *V*_max_ value obtained was 410 μmol SCN^-^(min^-1^(mg^-1^, yielding a *k*_cat_**of 64 s^-1^. The kinetic constants were about the same as those reported previously [9], where *K_m_* values for thiosulfate and cyanide of 4.6 and 27 mM, respectively, were found. The small differences observed may be due to the presence or absence of the His-6 tag, or due to differences in concentrations of thiosulfate employed in the two studies. Thiosulfate concentrations in the present study were above and below *K_m_* (0.5 – 5 mM), whereas Adams *et al*. used high concentrations of thiosulfate (10 – 50 mM).

As is the case for other rhodaneses, PspE also was capable of using a dithiol (dithiothreitol) as sulfur acceptor, ultimately releasing sulfide and oxidized dithiothreitol. For this reaction, the specific activity was about 300 U per mg of PspE, and the apparent *K_m_* for dithiothreitol was about 10 mM when using either 4 mM or 10 mM thiosulfate. Finally, purified PspE possessed very low mercaptopyruvate sulfurtransferase activity. With cyanide as the sulfur acceptor (thiocyanate detection), the ratio of rhodanese to MST activity (5 U/mg) was 40; with 2-mercaptoethanol as sulfur acceptor (pyruvate detection), the ratio of rhodanese to MST activity (0.2 U/mg) was 900.

The *E. coli* rhodanese GlpE is inhibited only weakly by certain anions [8]. Greater inhibition by anions was observed for PspE. Addition of sodium sulfate or sodium chloride at 0.25 M resulted in about 50% inhibition of rhodanese activity. Addition of sodium phosphate at the same concentration resulted in about 65% inhibition. Sulfite, one of the products of the rhodanese reaction, strongly inhibited PspE activity (1 mM sodium sulfite resulted in more than 50% inhibition of rhodanese activity, data not shown).

## DISCUSSION

Characterization of strains deficient in *pspE* and/or *glpE *established that the encoded single-domain rhodaneses are the major contributors of rhodanese activity in *E. coli*. Strains lacking both *pspE* and *glpE* had less than 5% of the activity found in wild-type strains. Lack of rhodanese activity had no apparent influence on the growth of *E. coli* under the various conditions tested. It was hypothesized that one of the single-domain rhodaneses might participate in trafficking of sulfur during biosynthesis of sulfur-containing cofactors. For example, the MoeB protein of *E. coli* lacks a rhodanese domain, and so might require a stand-alone rhodanese for sulfur trafficking during molybdopterin biosynthesis, as is the case for the human enzyme [12]. However, it is apparent that neither GlpE nor PspE is essential for molybdopterin synthesis, or for synthesis of other essential sulfur-containing cofactors. It is possible that one of the other sulfurtransferases present in *E. coli* cooperates with MoeB during molybdopterin synthesis. However, characterization of mutants deficient in each of the other rhodanese genes has not yielded further information regarding physiological function (unpublished data).

**Fig. (6):**
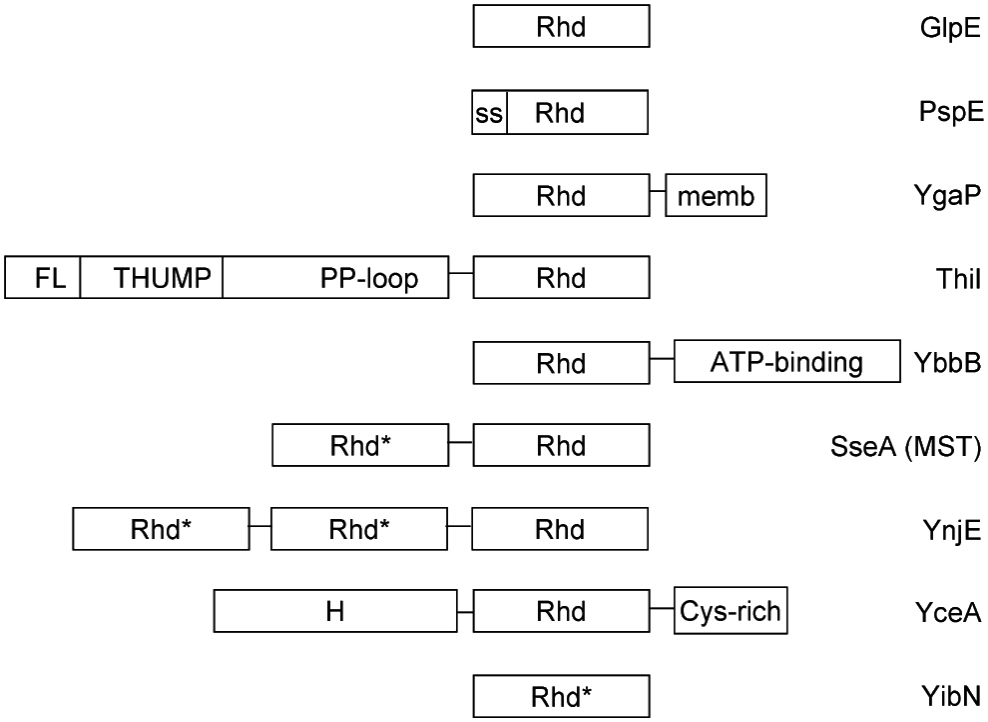
Schematic illustrating rhodanese paralogs found in *E. coli*. The Abbreviations used are: Rhd, rhodanese homology domain; Rhd*, rhodanese homology domain in which an aspartate replaces the conserved active site cysteine of Rhd; ss, cleaved signal sequence of PspE; memb; hydrophobic segment of YgaP; FL, THUMP and PP-loop are the ferredoxin-like domain, RNA-binding domain found in RNA base-modifying enzymes, and ATP pyrophosphatase domains, respectively, of ThiI [54]; ATP-binding, domain of tRNA 2-selenouridine synthase involved in nucleotide binding, tRNA interaction and modification; H, hypothetical domain of unknown function; Cys-rich, cysteine-rich domain of YceA.

*E. coli* and most other organisms possess multiple proteins with the rhodanese domain. Although the nine paralogs found in *E. coli* are easily identified based on their conserved sequence motifs, they are not very similar in sequence to one another, and some of their rhodanese domains are found fused to other unique protein domains (Fig. **6**). In addition, the proteins are found not only in the cytoplasm, but also in other subcellular locations (e.g., periplasm for PspE, cytoplasmic membrane for YgaP). Therefore, the different paralogs are likely to have defined, unique physiological roles. As pointed out in the Introduction, two of these proteins participate in modifying tRNAs (ThiI and YbbB), but the functions of the others must still be defined.

Results presented in this paper indicate that PspE is not likely to be an essential part of the phage shock regulon. Expression of rhodanese activity was not induced by stressors that result in induction of the other members of the *psp* operon (heat stress, osmotic stress), but instead was induced during growth on less preferred carbon sources such as glycerol. There are no obvious simple transcription terminators preceding *pspE*, and so it is likely that *pspE* expression also could be induced by signals that induce the *pspABCD* operon to its highest levels (filamentous phage infection, or in regulatory mutants). Microarray analysis in which mRNA levels in a *pspF* (positive regulator of *psp* gene expression) mutant were compared with those in a *pspA* (negative regulator) mutant strain revealed apparent co-regulation of *pspE* with the preceding genes of the operon [53]. Previous studies also concluded that PspE is regulated independently from the other members of the *psp* operon, since a short, *pspE*-specific transcript was observed that is thought to originate from an independent, σ^70^-dependent promoter presumably located within the 75-bp *pspD-pspE* intergenic region [22,38]. The premise that *pspE* is not an essential member of the *psp* regulon is also supported by the observation that among organisms in which the *pspABC* and *pspF* genes are conserved, the *pspD* and *pspE* genes have limited conservation [23]. 

Likewise, the present work demonstrates that *glpE* is not an essential part of the *glp* regulon, since the mutant strains grew normally on minimal medium containing glycerol. Our earlier work revealed that the main promoter for the *glpEGR* operon is not subject to specific regulation by GlpR [19], and analysis of completely sequenced bacterial genomes indicates that organization of *glpE*, *glpG* and *glpR* into an operon is not conserved. Therefore, it is possible that the context of *glpE* within the *glpEGR* operon of *E. coli* is fortuitous, and has no physiological relevance. 

The catalytic properties of native PspE were similar to those of PspE containing an N-terminal polyhistidine tag [9], and to those of previously characterized “accessible” rhodanese of *E. coli* [55]. In contrast, the properties of PspE were distinct from those of cytoplasmic rhodanese GlpE (Table **4**). The main distinction is that PspE has a much higher affinity for thiosulfate compared to GlpE. This property suggests that thiosulfate is in fact a physiological sulfur donor for PspE, which may not be the case for GlpE. Neither enzyme has a high affinity for cyanide, which indicates that these enzymes would not be effective for cyanide detoxification. Indeed, strains deficient in either or both enzymes were not more sensitive to cyanide, and overexpression of either enzyme did not confer resistance to cyanide (reference [9] and our unpublished results).

The various covalently modified forms of PspE detected by mass spectrometry are explained by the known mechanism of rhodanese catalysis, as well as by known reactions of compounds containing sulfur [56]. Rhodaneses catalyze the transfer of sulfur from thiosulfate by using a two step ping-pong mechanism, whereby a covalent enzyme-sulfur intermediate is formed on the active site cysteine of the enzyme during the first step:

E-SH + SSO_3_^2-^ → E-S-S^-^ + HSO_3_^- 			           ^(1)

During the second step, a thiophilic acceptor such as cyanide displaces the bound sulfur, returning the enzyme to its active form (E-SH):

E-S-S^-^ + CN^- ^→ E-SH + SCN^- 			           ^(2)

Presumably, PspE carrying a second sulfur atom is generated *via *further reaction of the enzyme-sulfur intermediate with another molecule of sulfur donor:

E-S-S^-^ + SSO_3_^2-^ → E-S-SS^-^ + HSO_3_^-		           ^(3)

The sulfonate form of the enzyme appears to be generated by reaction with thiosulfate, since its abundance increased following incubation of PspE2 with thiosulfate (reaction 4). Alternatively, sulfite generated during the rhodanese reaction would be capable of reaction with an oxidized, disulfide form of PspE (reaction 5):

E-S-S^-^ + SSO_3_^2-^ → E-S-SSO_3_^2-^ + HS^-^ 		       (4)

E-S-S-E + HSO_3_^-^ → E-S-SO_3_^2-^ + E-SH 		       (5)

The basis for activation of PspE by thiol reagents such as cysteine or dithiothreitol is that these reagents will not only reduce disulfide-linked PspE dimers (reaction 6), but should also react with the sulfonate carried by the active site cysteine or cysteine persulfide (reactions 7 and 8 [56]):

E-S-SO_3_^-^ + R-SH → E-S-S-R + HSO_3_^- 		           ^(6)

E-S-SSO_3_^-^ + R-SH → E-S-S-R + SSO_3_^2- 		           ^(7)

E-S-SSO_3_^-^ + R-SH → E-S-S-S-R + HSO_3_^- 		           ^(8)

The active form of the enzyme (E-SH or E-SSH) would be generated by intramolecular reduction of the mixed disulfide (in the case of R = dithiothreitol) or by reaction with a second molecule of cysteine (R-SH, reaction 9):

E-S-S-R + R-SH → E-SH + R-S-S-R (9)

It should be pointed out that the active form of the enzyme may be the cysteine persulfide form of the enzyme. The persulfide sulfur is apparently quite resistant to removal by cyanide, as demonstrated by the fact that very little sulfur was removed from fraction PspE2 (Fig. **4**) even though the persulfide derivative was the most abundant form of PspE found in this fraction (Table **3**). It has been observed that the persulfide forms of GlpE and RhdA are stable and may be the active forms of those enzymes [17,57]. 

The covalent modifications of the active site cysteine of PspE are likely to render the enzyme much less susceptible to oxidation or modification by thiol reagents. The stability of covalently modified PspE may be of physiological relevance, since it is present in the periplasmic space, an oxidizing environment. 

The physiological function of PspE remains to be determined. It is possible that PspE participates in trafficking of sulfur needed for formation or repair of sulfur-containing clusters of enzymes within the periplasm, as suggested earlier [9]. PspE should be suited for such a role, since it is capable of storing multiple sulfur atoms at each active site.

## Figures and Tables

**Table 1:** *E. coli* Strains

Strain	Description	Reference or derivation
MG1655	F^!^* ilvG rfb-50 rph-1 λ^!^*	[29]
BL21(DE3)	*hsdS gal (λ cI857 ind-1 Sam7 nin-5 lacUV5-T7* gene 1)	[30]
DH5αZ1	(*Ф80d lacZΔM15) endA1 recA1 hsdR17 supE44 thi-1 gyrA96 relA1 Δ(lacZYA-argF)U169 (λ att lacI^q^ tetR Sp^r^*)	[31]
CAG12028	MG1655 *zcj-233*::Tn*10*	[32]
TL524	MG1655 Δ(*lacZYA-argF)U169*	[33]
TST3	MC4100 malT::Tn10	[34]
BW25113	*lacI^q^ rrnB_T14_ ΔlacZ_WJ16_ hsdR514* *ΔaraBAD_AH33_ ΔrhaBAD_LD78_*	[35]
AL1	BW25113 ΔglpE::Km^r^ FRT (pKD46)	This work
HC1.1	BW25113 *ΔpspE*::Km^r^ FRT (pKD46)	This work
HC6.1	TL524 *ΔpspE*::Km^r^ FRT	P1(HC1.1 → TL524)
HC7.1	TL524 *ΔpspE*::FRT ^a^	FLP removal of Km^r^ from HC6.1
HC8.1	TL524 *ΔpspE::FRT ΔglpE*::Km^r^*FRT*	P1(AL1 → HC7.1)
JLD17201	TL524* ΔglpE*:: Km^r^*FRT*	P1(AL1 → TL524)
JLD17204	TL524 *ΔglpE::FRT ^a^*	FLP removal of Km^r^ from JLD17201
FA035	TL524 *ΔpspE::FRT ΔglpE::FRT^a^*	FLP removal of Km^r^ from HC8.1

^a^A 113-bp FRT “scar” remains following removal of the Km^r^ cassette by FLP recombinase [35].

**Table 2:** Comparison of Rhodanese Activities in Wild-Type and Sul-furtransferase-Deficient Strains

Strain	Genotype	Sp. act. (U/mg)^a^
TL524	Wild type	0.065
JLD17204	Δ*glpE::FRT*	0.066
HC7.1	Δ*pspE::FRT*	0.013
HC8.1	Δ*pspE::FRT * Δ*glpE:: Km^r^FRT*	0.003

^a^Cultures were grown in minimal glucose medium at 37°C and harvested at an OD_600_ of ~1.0. Specific activities were determined using crude extracts derived from sonicated cells and are expressed as units per mg protein. Substrate concentrations employed were 50 mM thiosulfate and 50 mM cyanide.

**Table 3:** Relative Abundances and Masses of PspE Species Found in Purified Fractions PspE1 and Ps

Enzyme form ^a^ (theoretical mass)	Enzyme Fraction					
	PspE1		PspE2		PspE2 ^b^ + SSO_3_^2-^	
	Abundance ^c^	Mass	Abundance	Mass	Abundance	Mass
E-SH (9427.7)	0.23	9427.9	0.6	9427.8	0.25	9427.6
E-SSH (9459.76)	0.57	9459.9	1	9460.1	0.65	9460.3
E-SSSH (9491.83)	1	9492.2	0.32	9491.9	0.71	9492.4
E-SSO_3_H (9507.76)	0.67	9507.9	0.6	9507.8	1	9507.9
E-SSSO_3_H (9539.83)	<0.1	--	<0.1	--	0.38	9540.5

^a^The species of PspE observed in the mass spectral analysis are consistent with those indicated, with E-SH being the unmodified form of PspE. E-SSH is PspE containing the cys-teine persulfide, E-SSSH is the enzyme with two additional sulfur atoms, E-SSO3H is the form with a sulfonate modification and E-SSSO3H is the persulfide form with a sulfonate modification. The theoretical masses shown in parentheses are those calculated based on the amino acid sequence of processed (secreted) PspE as deduced from the DNA sequence.

^b^An aliquot of fraction PspE2 was treated with 1 mM ammonium thiosulfate as described in “Methods.”

^c^The average abundances of the major peaks observed in the mass spectra, relative to the most abundant peak observed (designated 1.00), are shown. The molecular masses for each peak were calculated by averaging the masses obtained by deconvolution of the spectra observed for the +10, +11, +12 and +13 molecular ions.

**Table 4.** Comparison of PspE and GlpE Rhodaneses

Enzyme	*M_r _*(AA)	*k_cat _*(s^-1^)	*K_m_*, SSO_3_^2- ^(mM)	*K_m_*, CN^- ^(mM)
PspE	9,428 (85)	64	3.4	32
GlpE	12,082 (108)	115	78	17
